# Angiotensin II receptor I auto-antibodies following SARS-CoV-2 infection

**DOI:** 10.1371/journal.pone.0259902

**Published:** 2021-11-17

**Authors:** Yonghou Jiang, Fergal Duffy, Jennifer Hadlock, Andrew Raappana, Sheila Styrchak, Ingrid Beck, Fred D. Mast, Leslie R. Miller, William Chour, John Houck, Blair Armistead, Venkata R. Duvvuri, Winnie Yeung, Micaela Haglund, Jackson Wallner, Julie A. Wallick, Samantha Hardy, Alyssa Oldroyd, Daisy Ko, Ana Gervassi, Kim M. Murray, Henry Kaplan, John D. Aitchison, James R. Heath, D. Noah Sather, Jason D. Goldman, Lisa Frenkel, Whitney E. Harrington

**Affiliations:** 1 Center for Global Infectious Disease Research, Seattle Children’s Research Institute, Seattle, WA, United States of America; 2 Institute for Systems Biology, Seattle, Washington, United States of America; 3 Providence St. Joseph Health, Renton, Washington, United States of America; 4 Division of Biology and Biological Engineering, California Institute of Technology, Pasadena, California, United States of America; 5 Keck School of Medicine, University of Southern California, Los Angeles, California, United States of America; 6 Swedish Center for Research and Innovation, Swedish Medical Center, Seattle, Washington, United States of America; 7 Swedish Cancer Institute, Swedish Medical Center, Seattle, Washington, United States of America; 8 Department of Pediatrics, University of Washington, Seattle, Washington, United States of America; 9 Division of Allergy & Infectious Diseases, University of Washington, Seattle, Washington, United States of America; University Hospital Frankfurt, GERMANY

## Abstract

**Background:**

Coronavirus disease 2019 (**COVID-19**) is associated with endothelial activation and coagulopathy, which may be related to pre-existing or infection-induced pro-thrombotic autoantibodies such as those targeting angiotensin II type I receptor (**AT1R-Ab**).

**Methods:**

We compared prevalence and levels of AT1R-Ab in COVID-19 cases with mild or severe disease to age and sex matched negative controls utilizing multivariate logistic and quantile regression adjusted for comorbidities including hypertension, diabetes, and heart disease.

**Results:**

There were trends toward increased prevalence (50% vs. 33%, p = 0.1) and level of AT1R-Ab (median 9.8 vs. 6.1 U/mL, p = 0.06) in all cases versus controls. When considered by COVID-19 disease severity, there was a trend toward increased prevalence of AT1R-Ab (55% vs. 31%, p = 0.07), as well as significantly higher AT1R-Ab levels (median 10.7 vs. 5.9 U/mL, p = 0.03) amongst individuals with mild COVID-19 versus matched controls. In contrast, the prevalence (42% vs. 37%, p = 0.9) and level (both medians 6.7 U/mL, p = 0.9) of AT1R-Ab amongst those with severe COVID-19 did not differ from matched controls.

**Conclusions:**

These findings support an association between COVID-19 and AT1R-Ab, emphasizing that vascular pathology may be present in individuals with mild COVID-19 as well as those with severe disease.

## Introduction

Coagulopathy occurs frequently with severe disease from severe acute respiratory syndrome coronavirus 2 (**SARS-CoV-2**) infection [1]. Case series of patients admitted to the intensive care unit and autopsies describe high rates of both pulmonary embolism and small vessel inflammation and thrombosis in the lungs and other organs [2, 3]. A number of mechanisms that may mediate coronavirus disease 2019 (**COVID-19**)-associated coagulopathy have been proposed including direct endovascular damage, altered platelet function, and pre-existing or infection-induced pro-thrombotic auto-antibodies [1]. Pro-thrombotic anti-phospholipid antibodies have been described in some but not all patients with COVID-19 associated coagulopathy [4], and recent research suggests the potential for cross-reactive auto-antibodies following infection with as yet unidentified targets [5].

Amongst previously described anti-endothelial antibodies, those directed against the angiotensin II type 1 receptor (**AT1R-Ab**) are associated with essential hypertension [6], pre-eclampsia [7], and vascular rejection following renal transplant [8]. AT1R is part of an angiotensin II-driven signaling cascade that leads to increased blood pressure and inflammatory cytokine production; the pathway may be inhibited by angiotensin-converting enzyme 2, which also functions as the primary receptor for SARS-CoV-2 cell entry [9]. In particular, AT1R-activating auto-antibodies (**AT1R-AA**) directed against the second extracellular loop of AT1R are associated with pathology [7, 8], including elevated pro-inflammatory TNF-α and IL-6 cytokine levels and increased disease severity in pre-eclampsia models [7]. AT1R-AA are reported to induce expression of tissue factor by vascular smooth muscle which may trigger aberrant coagulation and clot formation [10]. In renal transplant recipients, AT1R-AA are associated with refractory rejection and malignant hypertension, as well as vasculopathy including arterial inflammation, endothelial activation, tissue factor expression, and thrombosis [8].

Multi-organ endothelial inflammation and increased cytokine production are strongly associated with COVID-19 disease severity and poor outcomes [2, 3, 11]. Endothelial damage may allow the binding of or trigger the development of anti-endothelial antibodies such as AT1R-Ab. COVID-19 has also been associated with elevated levels of IL-6 [12, 13], which in animal models may trigger the development of AT1R-Ab [14]. Alternatively, structural homology between epitopes of the SARS-CoV-2 Spike and the second extracellular loop of AT1R might lead to the development of cross-reactive antibodies. These possibilities led us to consider AT1R-Ab as a potential mediator of COVID-19-associated coagulopathy and disease. If directed against the second extracellular domain of AT1R, such antibodies might trigger hypertension, inflammation including cytokine storm, and pulmonary edema as seen in severe COVID-19. In addition, AT1R-Ab antibodies might contribute to further endothelial dysfunction, tissue factor expression, and hypercoagulability. We therefore tested the hypothesis that COVID-19 infection is associated with a higher prevalence and levels of AT1R-Ab compared to uninfected controls and assessed whether AT1R-Ab in SARS-CoV-2 negative individuals binds to SARS-CoV-2 Spike trimer.

## Materials and methods

### Cohorts and samples

The present study included mild and severe COVID-19 cases derived from three parent cohorts, along with age and sex matched controls without a history of COVID-19. Mild COVID-19 cases were from the Seattle Children’s SARS-CoV-2 Recovered Cohort and the Seattle Children’s SARS-CoV-2 Prospective Cohort both approved by Seattle Children’s Hospital Institutional Review Board (**IRB**) (STUDY00002048; STUDY00002434), with study specimens collected from ≥14 days and <60 days from onset of COVID-19 symptoms (**Table 1**). Inclusion criteria from these two cohorts included a history of positive PCR for SARS-CoV-2 and completion of isolation following acute infection, and exclusion criteria included pregnancy, history of malignancy or autoimmune disease, and weight less than 110 pounds. All participants recovered at home. Severe COVID-19 cases were from participants in the Swedish-Institute for Systems Biology Novel Coronavirus (**INCOV**) Biobank, approved by Providence St. Joseph Health IRB (STUDY2020000175), with samples from ≥7 days from symptom onset. Inclusion criteria for the parent protocol included a positive PCR for SARS-CoV-2 and the ability to consent and participate, and exclusion criteria included hematocrit <25 and cognitive impairment. The severe COVID-19 cases had maximum WHO COVID-19 scores between 4 and 8 (median 5), and six individuals died from complications of COVID-19. Human leukocyte antigen (**HLA**) typing by direct sequencing had previously been conducted for the INCOV cohort. For the primary analysis, cases were matched 1:1 to SARS-CoV-2 negative controls by age and sex. SARS-CoV-2 negative individuals were derived from the Children’s SARS-CoV-2 Prospective Cohort. At the time of blood collection (March to July 2020), SARS-CoV-2 negative individuals reported no prior history of SARS-CoV-2-like illness, were nasal specimen PCR negative for SARS-CoV-2, and were SARS-CoV-2 antibody negative on the SCoV-2 Detect™ IgG and IgM ELISAs (InBios, Seattle, WA). All participants provided written informed consent.

**Table 1:** Cohort demographics by COVID-19 status.

	All COVID-19 Controls & Cases			Mild COVID-19 Controls & Cases			Severe COVID-19 Controls & Cases		
Demographics	Controls	Cases	p-value	Controls	Cases	p-value	Controls	Cases	p-value
**Number**	48	48		29	29		19	19	
**Age, years, median [range]**	46 [24–73]	45 [24–88]	0.8	41 [24–71]	40 [24–74]	0.8	62 [38–73]	64 [38–88]	0.6
**Female, n (%)**	29 (60%)	29 (60%)	1	22 (76%)	22 (76%)	1	7 (37%)	7 (37%)	1
**Days from 1st symptom, median [range]**		22 [7–59]			31 [14–59]			13 (7–37)	

### Structural modeling

To assess the biologic plausibility that SARS-CoV-2 possesses antigenic sites that elicit AT1R-Ab that could cross-react with endothelium, searches were performed to identify regions of structural homology between AT1R and SARS-CoV-2 Spike protein. The UCSF Chimera MatchMaker tool was used, which aligns structures based on pairwise sequence alignment followed by 3D structural alignment using residue Cα positions. Spike protein monomer [PDB: 6VXX] was used as the SARS-CoV2 reference structure compared to human AT1R protein [PDB: 4YAY].

### Anti-AT1R antibody screening

The concentration of AT1R-Ab in plasma was measured with a quantitative ELISA (Celltrend, Luckenwalde, Germany) using the entire AT1R protein, with assays performed according to the manufacturer’s instructions. In brief, standards and diluted samples (1:100) were added to AT1R-precoated microtiter plates and incubated for 2 h at 4°C. After washing, AT1R-Ab was detected with HRP-labelled anti-human IgG antibody followed by enzymatic substrate reaction. Samples were tested in duplicate. Optical densities (**OD**) were converted into concentrations by comparison to a standard curve and mean concentration was used to define a sample as positive when ≥10 U/mL and negative when <10 U/mL [15].

### Anti-SARS-CoV-2 Spike, receptor binding domain, and nucleocapsid protein ELISAs

In order to investigate potential cross-reactivity between AT1R-Ab and SARS-CoV-2 Spike, we tested plasma from AT1R-Ab positive, SARS-CoV-2 negative individuals and randomly selected AT1R-Ab negative, SARS-CoV-2 negative individuals for reactivity against SARS-CoV-2 Spike trimer (including both S1 and S2 domains), as previously published [16]. We hypothesized that potential cross-reactive antibodies might bind SARS-CoV-2 Spike with lower avidity, therefore, we tested plasma at an initial dilution of 1:10 followed by serial dilutions by a factor of two. To assess for potential cross-reactivity induced by prior non-SARS-CoV-2 coronavirus infection, we additionally tested these control subjects against the receptor binding domain (**RBD**) in S1 and the nucleocapsid protein (**NP**) using a similar ELISA format. Endpoint titers were defined as the reciprocal of plasma dilution at OD 0.1 at a wavelength of 450 nm after the subtraction of plate background. Positive antibody responses were defined at a titer greater than or equal to 1:50 for each antigen.

### Statistical analysis

Based on prior literature reporting AT1R-Ab levels in healthy controls [17] and in those with arterial injury following renal transplant [18], we estimated that a difference in AT1R-Ab level between our cases and controls of 5 U/mL (15 versus 10 U/mL, standard deviation 5 U/mL) would be clinically meaningful. Using a two-sided t-Test with an α = 0.05, we estimated that we would need to include at least 16 cases and controls to detect a difference between groups with 80% power.

AT1R-Ab prevalence and levels were first compared between all cases versus all controls. In secondary analysis, participants with mild or severe COVID-19 were compared separately to their age and sex matched controls. Comorbidities including history of hypertension, diabetes, and heart disease were colinear, so a composite comorbidity score was generated for each individual by tallying the number of comorbid conditions. The prevalence of AT1R-Ab (negative versus positive) was compared between groups using multivariate logistic regression, and AT1R-Ab levels were compared between groups using multivariate quantile regression on the median, with models adjusted for the comorbidity score. Distribution of HLA and SARS-CoV-2 antibody responses by AT1R-Ab status were compared with the Chi-square test. Significance was defined as p≤0.05, whereas trend was defined as p≤0.1.

## Results

### Association between AT1R-Ab and SARS-CoV-2 infection

Participants with mild versus severe COVID-19 were younger and more likely to be female (**Table 1**), consistent with prior reports of older age and male sex as risk factors for severe COVID-19.

**Fig 1:**
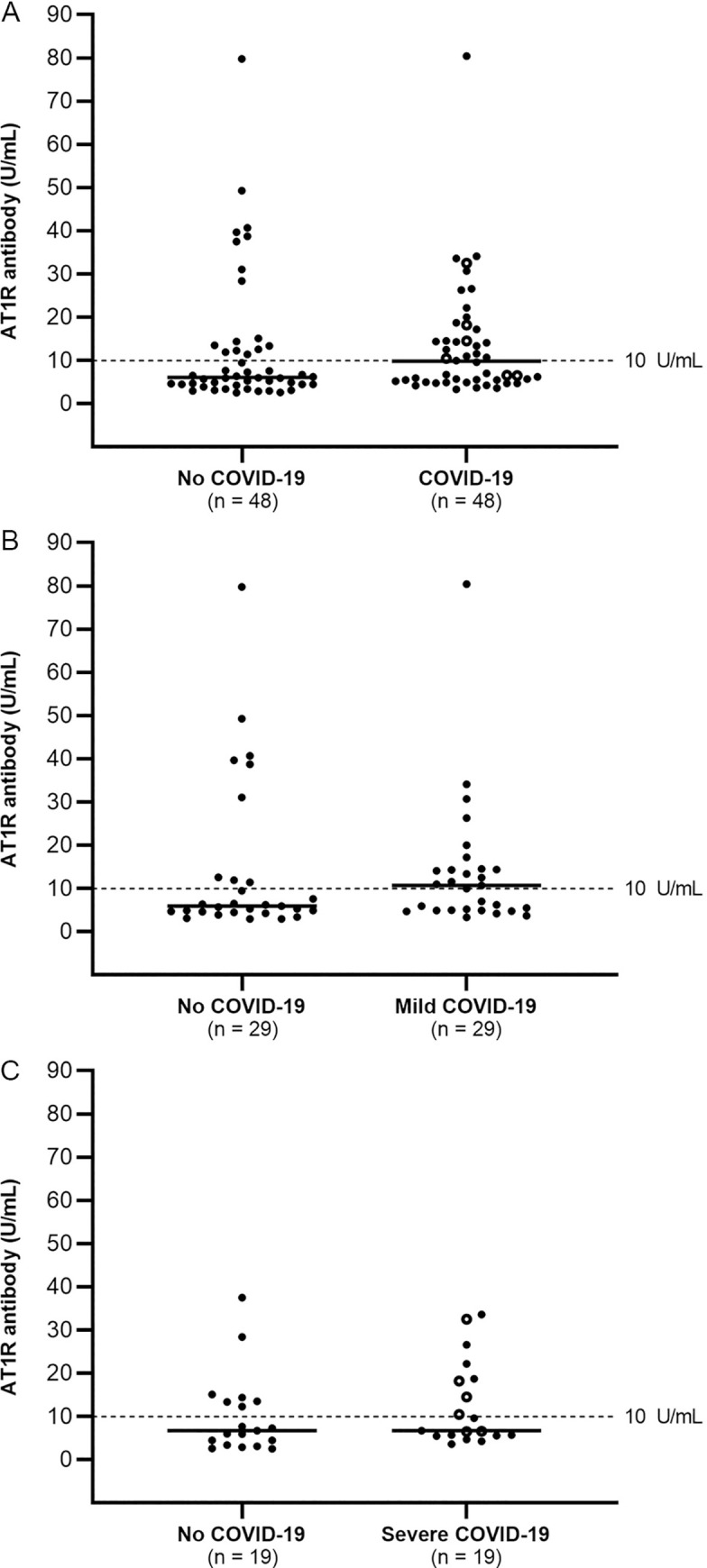
Association between COVID-19 and AT1R-Ab. AT1R-Ab levels < 10U/mL were defined as negative and ≥ 10 as positive (dashed line) [15]. Solid lines indicate medians. Open circles are cases with known thrombosis. (**A**) There was a trend toward higher level of AT1R-Ab in all COVID-19 cases versus controls (median 9.8 vs 6.1, p = 0.06) and (**B**) significantly higher levels amongst the subset of mild versus matched controls (median 10.7 vs 5.9, p = 0.03). (**C**) The levels in severe cases and matched controls did not differ (both medians 6.7U/mL, p = 0.9).

There was a trend toward increased prevalence of AT1R-Ab in COVID-19 cases versus controls (50% vs. 33%, p = 0.1) as well as in the subset of mild cases versus controls (55% vs. 31%, p = 0.07) (**Fig 1A and 1B**). In contrast, the prevalence of AT1R-Ab was similar among severe cases versus controls (42% vs. 37%, p = 0.9) (**Fig 1C**). Similarly, there was a trend toward higher median AT1R-Ab level in all COVID-19 cases versus controls (median 9.8 vs. 6.1 U/mL, p = 0.06), and significantly higher AT1R-Ab level amongst the subset of mild COVID-19 cases versus matched controls (median 10.7 vs. 5.9 U/mL, p = 0.03). AT1R-Ab levels in severe COVID-19 cases versus controls did not differ (both medians 6.7 U/mL, p = 0.9) (**Fig 1**).

### Association between AT1R-Ab, thrombotic events, and HLA-type amongst severe COVID

Within the severe COVID-19 group, we tallied six participants with thrombotic events. These included one participant with a myocardial infarction and one with a pulmonary embolism, both with high levels of AT1R-Ab (33 and 18 U/mL respectively), and four participants with deep venous thrombosis with two positive for AT1R-Ab (11 and 14 U/mL). Individuals with thrombosis had a non-significantly higher prevalence of AT1R-Ab relative to individuals without thrombosis (67% vs 31%, p = 0.2). Similarly, while the median level of AT1R-Ab was higher in those with thrombosis, this was not statistically significant (12.5 vs. 5.7 U/mL, p = 0.4).

AT1R-Ab have previously been associated with the HLA DRB1*04 group [19]. There was a trend toward a higher prevalence of DRB1*04 allele carriage in individuals positive versus negative for AT1R-Ab (50% vs. 18%, p = 0.1) (**S1 Table**).

### Structural homology between AT1R and SARS-CoV-2 Spike and assessment of antibody cross-reactivity

**Fig 2:**
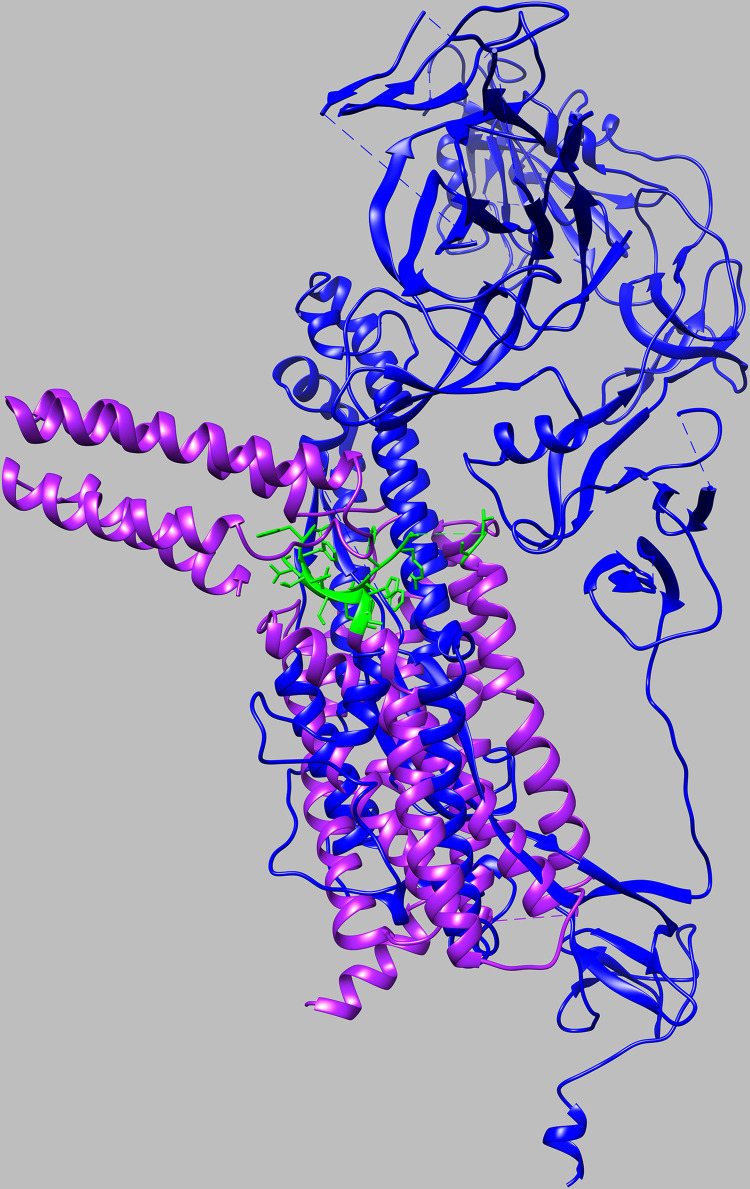
Structural overlap between SARS-CoV-2 Spike and AT1R. The structures of SARS-CoV-2 Spike (S1 and S2) and AT1R were aligned to assess potential homology. Overlap was identified between SARS-CoV-2 Spike (blue) in the S2 domain with the six-membered alpha-helical bundle of AT1R (purple), including the second extracellular domain (green). The root-mean square deviation over all 237 aligned residue pairs was 35.6 Å.

**Fig 3:**
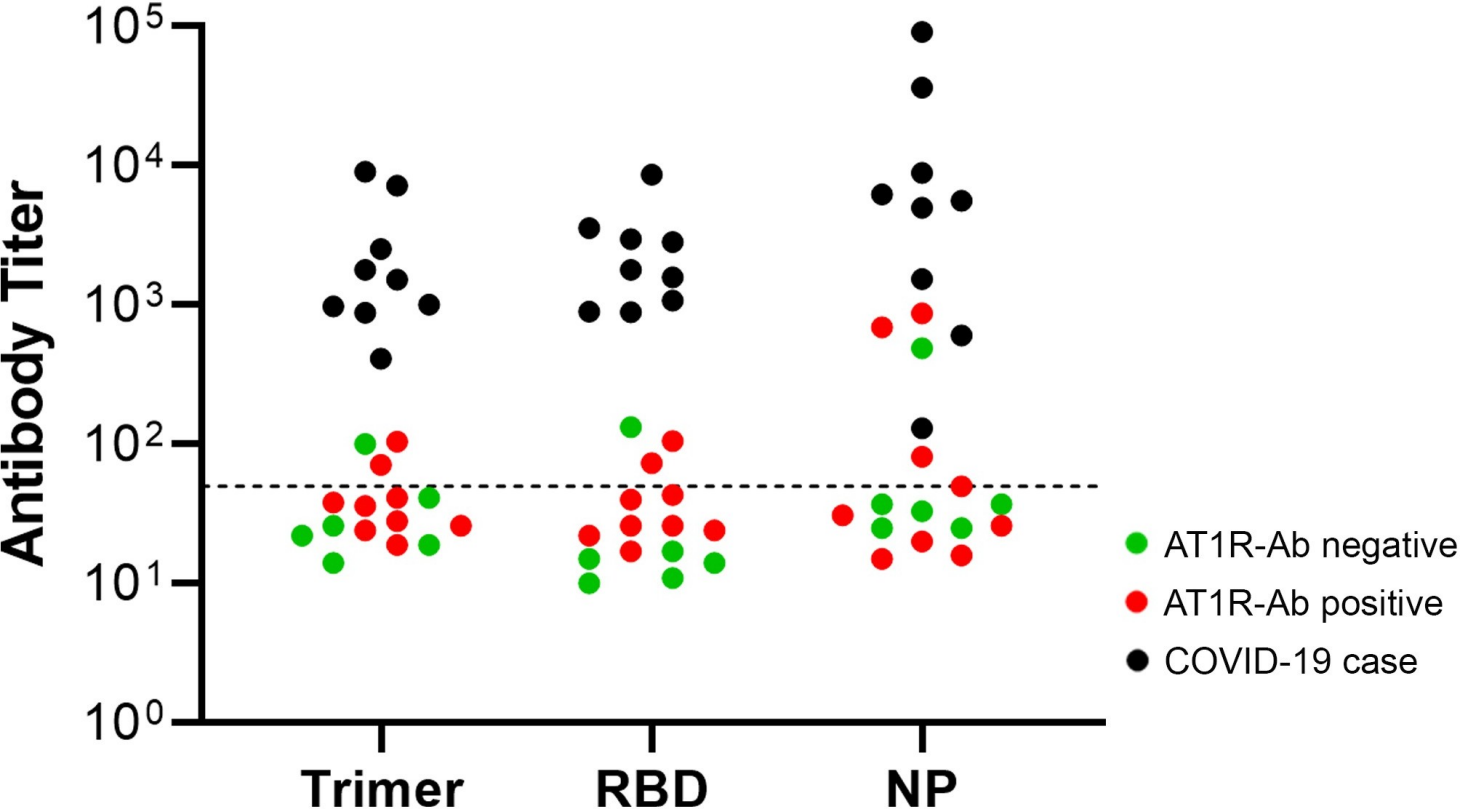
Assessment of SARS-CoV-2 protein immune reactivity by AT1R-Ab status in SARS-CoV-2 negative individuals and COVID-19 cases. ELISA for SARS-CoV-2 Spike trimer, RBD, and NP. Green: AT1R-Ab negative, SARS-CoV-2 negative individuals. Red: AT1R Ab positive, SARS-CoV-2 negative individuals. Black: COVID-19 cases. Endpoint titers were defined as the reciprocal of plasma dilution at OD 0.1 read at 450 nm after the subtraction of plate background. Positive threshold was defined as 1:50 and is indicated with dashed line. There was no clear relationship between AT1R-Ab status amongst SARS-CoV-2 negative individuals and immunoreactive pattern against SARS-CoV-2 proteins.

Potential cross-reactivity of antibodies between SARS-CoV-2 Spike and AT1R was predicted based on our finding of structural homology between the aligned proteins in the S2 domain of the SARS-CoV-2 Spike and the six-membered alpha-helical bundle of AT1R, including the second extracellular domain targeted by AT1R-AA (**Fig 2**). To test the hypothesis that AT1R-Ab cross-reacts against SARS-CoV-2 Spike, in particular the S2 domain, nine AT1R-Ab positive, SARS-CoV-2 negative individuals and six randomly selected AT1R-Ab negative, SARS-CoV-2 negative individuals were tested for reactivity against SARS-CoV-2 Spike trimer by ELISA. AT1R-Ab status in the SARS-CoV-2 negative individuals was not associated with reactivity to SARS-CoV-2 Spike trimer (17% AT1R-Ab negative vs. 22% AT1R-Ab positive, p = 0.8) In addition, some AT1R-Ab positive individuals demonstrated low level reactivity against RBD (17% AT1R-Ab negative vs. 22% AT1R-Ab positive, p = 0.8) and NP (17% AT1R-Ab negative vs. 44% AT1R-Ab positive, p = 0.3), with levels lower than COVID-19 cases (**Fig 3**).

## Discussion

Here we describe the prevalence and levels of AT1R-Ab amongst individuals with COVID-19 versus age and sex matched controls. We identified a trend toward increased prevalence and levels of AT1R-Ab in all COVID-19 cases versus controls, as well as significantly higher median AT1R-Ab levels amongst individuals with mild COVID-19 versus their matched controls. Our findings amongst individuals with mild COVID-19 are consistent with a recent report of increased levels of AT1R-Ab in individuals with COVID-19 versus healthy controls [20]. In contrast, we did not identify a difference between individuals with severe COVID-19 and their matched controls. Two recent publications compared AT1R-Ab in mild versus severe COVID-19, reporting higher levels in those with severe disease, however these analyses did not adjust for differences in demographic characteristics or comorbidities by COVID-19 severity, which may have been associated with higher baseline AT1R-Ab levels [15, 20]. Alternatively, the severe COVID-19 cases in our study were sampled relatively earlier in their disease course compared to our mild COVID-19 cases, a product of the parent study designs, and AT1R-Ab may have developed in the former individuals later in their disease course at a time when specimens were not available for testing.

Amongst severe COVID-19 cases, where HLA typing was available, half of individuals with AT1R-Ab carried a DRB1*04 allele [19]. Of note, DRB1*04:01 and DRB1*04:05 are both associated with increased risk of autoimmune disease [21]. These individuals may have had pre-existing AT1R-Ab not associated with their SARS-CoV-2 infection. Alternatively, when infected with SARS-CoV-2, individuals with this allele may have had a higher risk of developing AT1R-Ab in the setting of endothelial damage. We are not able to dissociate these possibilities given the cross-sectional nature of our sampling at the time of COVID-19.

We identified a region of potential structural homology between AT1R and the S2 domain of SARS-CoV-2 Spike; however, among SARS-CoV-2 negative individuals there was no clear association between AT1R-Ab reactivity and low-level reactivity against SARS-CoV-2 Spike trimer, arguing against cross-reactivity as a driver of AT1R-Ab development. In addition, some SARS-CoV-2 negative individuals reacted against RBD and NP, suggesting that the reactivity we detected may represent cross-reactive antibodies from prior endemic coronavirus infections [22]. It is possible but less likely that these individuals had experienced an unrecognized SARS-CoV-2 infection given their commercial antibody negative status and the date of their enrollment early in the pandemic when the prevalence of infection remained low in the greater Seattle area. These data emphasize alternative potential mechanisms for the induction of AT1R-Ab following COVID-19, including endothelial damage [2, 3, 11], inflammation or cytokine (particularly IL-6) triggered induction [12–14], or genetic predisposition such as carriage of the DRB1*04 allele [19].

Our study was limited by small sample size, particularly amongst our severe COVID-19 cases. In addition, as we did not have pre-infection samples from cases that would have allowed us to determine whether AT1R-Ab were pre-existing or developed following SARS-CoV-2 infection. Further, we were not able to determine whether AT1R-Ab positive, SARS2-CoV-2 negative individuals are at risk of developing more severe COVID-19 symptoms if infected.

An improved understanding of the mechanisms driving COVID-19-associated coagulopathy could point to therapies to lessen COVID-19 morbidity and mortality. Here we identified an association between AT1R-Ab and COVID-19 in individuals with mild symptoms, suggesting a risk for vascular pathology. Future research should explore whether AT1R-Ab are a pre-existing risk factor for symptomatic COVID-19 or are induced by the infection, as well as further elucidate the role of AT1R-Ab in hypercoagulability associated with COVID-19.

## Supporting information

S1 Table Human leukocyte antigen DRB1 allele by AT1R-Ab status amongst those with severe COVID-19. (DOCX)

S1 File Primary data for analysis of association between COVID-19 and AT1R-Ab status. (XLSX)

S2 File Primary data for analysis of association between AT1R-Ab status and anti-SARS-CoV-2 IgG levels amongst SARS-CoV-2 negative individuals. (XLSX)
